# The quantum hypercube as a k-mer graph

**DOI:** 10.3389/fbinf.2024.1401223

**Published:** 2024-09-12

**Authors:** Gustavo Becerra-Gavino, Liliana Ibeth Barbosa-Santillan

**Affiliations:** Doctorado en Technologías de Información, Universidad de Guadalajara, Centro Universitario de Ciencias Económicas Administrativas, Zapopan, Jalisco, Mexico

**Keywords:** k-mer graph, coined quantum walk, quantum search, quantum computing with python, qiskit, quantum register initialization

## Abstract

The application of quantum principles in computing has garnered interest since the 1980s. Today, this concept is not only theoretical, but we have the means to design and execute techniques that leverage the quantum principles to perform calculations. The emergence of the quantum walk search technique exemplifies the practical application of quantum concepts and their potential to revolutionize information technologies. It promises to be versatile and may be applied to various problems. For example, the coined quantum walk search allows for identifying a marked item in a combinatorial search space, such as the quantum hypercube. The quantum hypercube organizes the qubits such that the qubit states represent the vertices and the edges represent the transitions to the states differing by one qubit state. It offers a novel framework to represent k-mer graphs in the quantum realm. Thus, the quantum hypercube facilitates the exploitation of parallelism, which is made possible through superposition and entanglement to search for a marked k-mer. However, as found in the analysis of the results, the search is only sometimes successful in hitting the target. Thus, through a meticulous examination of the quantum walk search circuit outcomes, evaluating what input-target combinations are useful, and a visionary exploration of DNA k-mer search, this paper opens the door to innovative possibilities, laying down the groundwork for further research to bridge the gap between theoretical conjecture in quantum computing and a tangible impact in bioinformatics.

## 1 Introduction

This paper embarks on a journey through quantum computing basics, providing readers with a foundational understanding of quantum mechanics, qubits, and quantum algorithms. It then delves into quantum software stacks, elucidating the essential tools, programming languages, and development environments that drive quantum computing’s practical applications. Moving forward, it explores the coined quantum walk search, unraveling the intricate algorithm’s potential applications in fields such as combinatorial problems. Shifting gears, the paper investigates DNA 2-bit Encoding, a cutting-edge approach to data storage, and discusses the practical implications and prospects of this novel technology. Lastly, it presents a technique to input DNA patterns into a quantum register to execute a coined quantum walk search for DNA pattern matching. It highlights the unique research objectives, methodologies, and results at this paper’s heart, promising to contribute to the ongoing dialogue in these exciting fields.

### 1.1 DNA 2bit encoding

The DNA genetic code is based on the monomer nucleotides {T,C,A,G} . From the point of view of information, this set is, in fact, a 4-symbol alphabet, and it is representable by 2 bits in a binary number system (Nemzer, 2017). One possible mapping to represent genetic code in binary format is {T↔00,C↔01,A↔10,G↔11} . This mapping is used in the **.2bit** file format, 2bit, to encode DNA sequences is used in the current work.

### 1.2 K-mer sequencing

In dealing with comparisons and searching, a technique used in genomics to analyze DNA data breaks down DNA sequences in fragments of **k**-lenght of monomers (Langmead, 2016). These fragments are named according to the number of monomers in the fragment. If the number of monomers **k** is 1, the k-mer is called a 1-mer. If the number of monomers **k** is 2, the k-mer is called a 2-mer, and so forth. Examples of 2-mer DNA fragments are **CA** and **GC**.

### 1.3 Quantum computing basics

While the concepts in quantum mechanics have been around for about a century (Born, 1926), it was in the 1980s that those concepts were theorized as options in the computer science disciplines (Benioff, 1982). Applying these quantum mechanics principles to computing is now known as quantum computing (Steane, 1998). Over the years, quantum computing has evolved from a theoretical hypothesis into a tangible reality. In the contemporary landscape of technological advancement, the theoretical underpinnings of quantum computing have transformed into practical methodologies that allow us to execute techniques reliant on quantum principles for complex computations.

The basic unit of information in quantum computing is the qubit (Schumacher, 1995). While a binary bit can only be in a state of 0 or 1, the qubit has the property that it can be in a combined state of |0〉 or |1〉 (read ket 0 or ket 1). This property is called superposition. The wave function in Equation 1 expresses the general qubit state, |ψ〉 . The qubit state notation is taken from the Dirac bra-ket notation (Dirac, 1939).

$$|\psi \rangle =\alpha |0\rangle +\beta |1\rangle ,\;where\;|0\rangle =\left[\begin{array}{c} 1\\ 0 \end{array}\right],\;and\;\left|1\right\rangle =\left[\begin{array}{c} 0\\ 1 \end{array}\right]$$ (1)

One fundamental difference between a binary computer and a quantum computer is that measuring the binary bit state does not alter its state, whereas in quantum computing, measuring a qubit collapses it into a pure state |0〉 or |1〉 . The probability that a qubit will collapse into a |0〉 or |1〉 is given by Equation 2 where α and β are complex numbers capturing the amplitudes of the state in Equation 1.

$$|\alpha {|}^{2}+|\beta {|}^{2}=1$$ (2)

Qubits perform calculations using quantum gates or operators to manipulate the qubit states. One such gate is the Pauli-X gate, represented by the
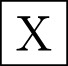
symbol (Nielsen and Chuang, 2000). This gate has the property of flipping the qubit state. If the qubit is in a |0〉 state the new state becomes |1〉 and *vice versa*. The transformation matrix in Equation 3 defines the Pauli-X gate. 

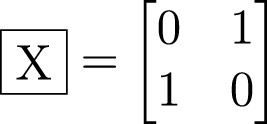

(3)



The
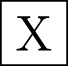
gate is a unitary operator; it is reversible. It is also applied to only one qubit. Other gates may be applied to a set of qubits. For instance, the CNOT gate defined in Equation 4 is one of the gates used to entangle two qubits (Hughes et al., 2021). The first is the control qubit; the other is the target qubit. With this gate, the qubits are set to interact between them. 

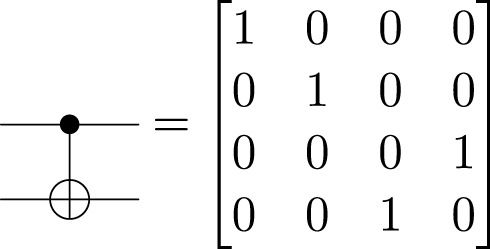

(4)



With both superposition and entanglement, qubit interference may be leveraged to perform computation. Since the qubit status is based on the quantum wave function, when two different qubits are entangled and subject to operators, their amplitudes will interact constructively or destructively. This phenomenon allows for computations beyond the capability of binary computing.

### 1.4 Quantum software stacks

Since the conception of the quantum computing concept, human ingenuity has been at work to explore the potential of this new computing paradigm. Quantum computing may increase cybersecurity (Bova et al., 2021), or break widely used cybersecurity technologies such as public key cryptography (Mavroeidis et al., 2018). It also may be used to speed up searching for a marked item in unstructured data through a quantum search. Since quantum computing has a promising outlook, companies worldwide are interested in facilitating quantum computing for research and commercial use through Quantum Software Stacks (QSS) (Wang et al., 2021). Google provides Cirq, Rigetti PyQuil, and IBM provides the Qiskit. The current work was researched, developed, and executed using IBM’s Qiskit QSS.

### 1.5 IBM quantum platform

**FIGURE 1:**
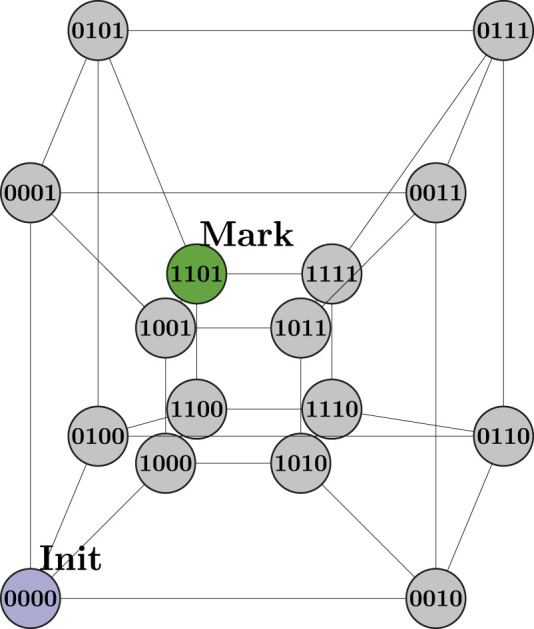
Hypercube with 4-bit nodes.

The IBM Quantum Platform, formerly known as the IBM Quantum Experience (Cross, 2018), is an open platform intended to ease the work of designing, developing, and running quantum circuits. Anyone interested may create these circuits through the Quantum Composer (Lehka et al., 2022), a cloud-based visual development environment. They also may be written in OpenQASM (Cross et al., 2017), an assembly-like computer language. Another familiar option is to write the quantum circuits using Python programming with the Qiskit (Qiskit contributors, 2023), modules installed. Qiskit allows for different quantum system backends to be used, both simulators and actual quantum processors with limited access. In addition, educational materials, such as the Qiskit Textbook (various authors, 2023), demonstrate tools available to create quantum algorithms. In this book, the coined quantum walk search algorithm (Wanzambi and Andersson, 2021), is implemented to search for a marked node in a tesseract, a hypercube with four dimensions as shown in Figure 1. This tesseract is built within the QuantumCircuit instance pointed by the **circuit** variable with the following Python code:

# Shift operator function for 4d-hypercube

def shift_operator(circuit) :

for i in range (0,4) :

circuit.x(4)

if i % 2 == 0 :

       circuit.x(5)

circuit.ccx(4,5,i)

This qubit arrangement allows us to represent the $2^{4}$ vertices in the hypercube with only four qubits. This is, in fact, an exponential information density.

### 1.6 The coined quantum walk search

The coined quantum walk search is a search algorithm targeted at unstructured databases. This search algorithm employs a quantum version of classical random walks executed on Markov chains (Shenvi et al., 2003; Boettcher et al., 2015). In the quantum version of the random walk, the walker evaluates several paths on the graph simultaneously through the superposition of states of the coin operator. The shift operator then takes the step influenced by the coin state. The phase estimation serves as the state evaluation tool to determine if a state is the search target. The coin is a set of qubits used to evaluate the walker’s next step. The coined quantum walk search demonstrated in the Qiskit textbook in chapter 3.10 uses 11 qubits. Four are used as the theta qubits for phase estimation, four for the tesseract nodes, two for the Grover’s coin, and one as an auxiliary (ancilla) qubit.

The coined quantum walk search stands out as a particularly promising paradigm. It holds the potential to transcend its theoretical origins and address an extensive array of problems, spanning an impressive spectrum of applications. Among these applications are solutions to combinatorial (Bova et al., 2021), problems where the search space is all the combinations of a finite set of symbols. DNA pattern matching belongs to this type of problem. In bioinformatics, DNA pattern matching and prediction plays such an important role that brilliant minds have designed practical algorithms to leverage traditional computing (Rahate and Chandak, 2018; Neamatollahi et al., 2020); and even advanced deep learning model techniques such as the Convolution Autoencoder (Guo et al., 2024). Poising our attention towards quantum computing, the quantum hypercube, with its exponential information density, also enables the prospect to execute the coined quantum walk search for a marked state.

### 1.7 The quantum hypercube as a K-mer graph

The current work researches a technique to encode DNA information to input it to a quantum computer and provide a target k-mer in the quantum hypercube search space for a coined quantum walk search algorithm to find. The coined quantum walk search is executed with each of the 16 possible combinations as a starting node. In addition, the 11-qubit quantum register is tested with all the possible initialization states. Each initialization state is executed with each of the 16 possible target nodes. The results generated are analyzed to provide insights into the effects of initializing the 11-qubit quantum register on the execution of the coined quantum walk search. The information is useful for peeking into the possibilities of leveraging the quantum hypercube as a k-mer graph to perform DNA pattern matching.

## 2 Materials and methods

### 2.1 Development platform

The IBM Quantum Software Platform facilitates the use of a quantum computer through the use of Python modules. These modules implement potent methods to build up and execute quantum circuits. The two packages used for the experiments in this research are the qiskit==0.39.0 and qiskit_ibm_provider==0.2.0 . Specifying these packages to be installed through the **pip** package manager also installs other packages as dependencies. Another advantage of using the Qiskit framework is that the circuits created will run as long as a compatible backend is available.

### 2.2 Loading DNA binary data into a quantum circuit

When using the Qiskit QSS, a QuantumCircuit instance is initialized to a $\left|0\right\rangle$ state by default. However, the Qiskit QuantumCircuit Python class allows for each qubit in instances of n-qubits to be initialized to a target state of $\left|0\right\rangle$ or $\left|1\right\rangle$ by passing a string of n-length of 0’s and 1’s as a parameter. The right-most character is applied to the first qubit from top to bottom, following a right-to-left and top-to-bottom order. This simplified initialization method is more familiar to classical programmers since this initialization string is, in fact, a binary string. For an 11-qubit circuit, an initialization string may take the form **“01001100010”**. When the method to initialize a quantum circuit object is called, the circuit is modified by adding
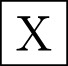
gates to rotate the qubits from a $\left|0\right\rangle$ state to a $\left|1\right\rangle$ state to achieve the desired circuit state.

The corresponding Python code with the modules installed and imported into the program is:

...

from qiskit import QuantumCircuit, execute, Aer, IBMQ, QuantumRegister, ClassicalRegister

...

circuit.initialize( “01001100010” , circuit.qubits)

...

**FIGURE 2:**
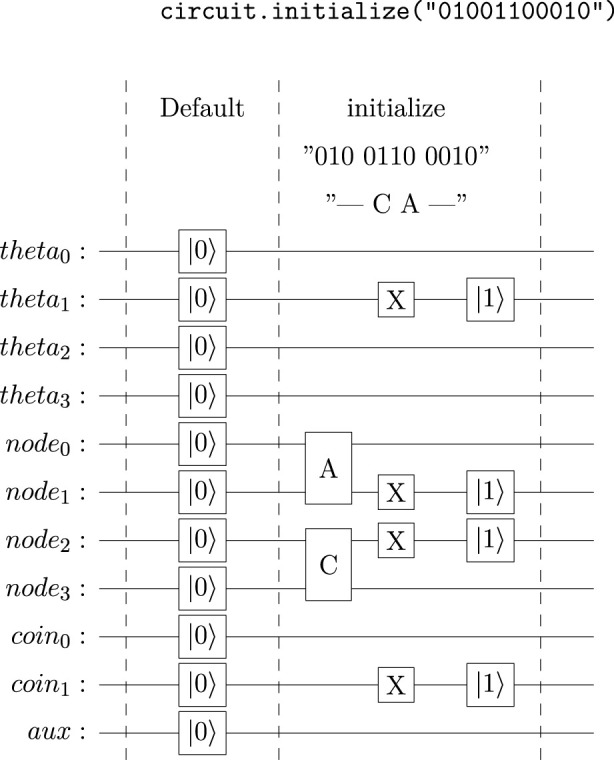
IBM Quantum Platform Register with the initialization string 01001100010 applied.

After calling the **circuit. initialize** method, the circuit is modified to set the quantum register into the specified state before executing the circuit. Thus, applying **“01001100010”** as the circuit initialization string to the coined quantum walk modifies the beginning of the circuit as illustrated in Figure 2.

The  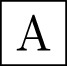 and  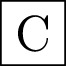 symbols in Figure 2 are not quantum gates. These two symbols illustrate how the quantum circuit is initialized to a desired tesseract node and what that node “0110” represents in a .2bit mapping for DNA sequences. Notice that the encoding is mapped from left to right to stay consistent with the Qiskit QuantumCircuit initialization string pattern.

### 2.3 The DNA hypercube space

**FIGURE 3:**
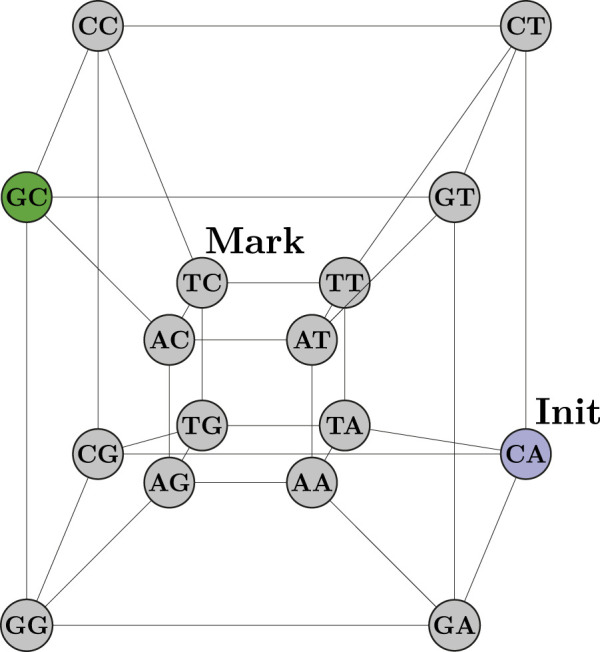
Hypercube with 2-mer DNA pattern nodes.

After the initialization method is called, the coined quantum walk implementation presented in the Qiskit Textbook is used to find the marked node in a hypercube with 4-bit vertices. These 4 bits represent two-letter DNA patterns, also called 2-mer substrings. This way, the hypercube in Figure 1 becomes the hypercube in Figure 3.

### 2.4 The coined quantum walk search circuit

**FIGURE 4:**
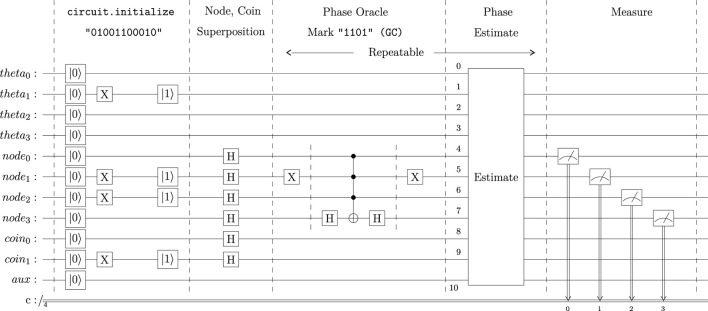
Complete Coined Quantum Walk Circuit with the CA DNA pattern provided as the initialization node and the node GC as a marked pattern.

The coined quantum walk implementation has three parts: A set of Hadamard gates applied to the node and coin qubits to set them into a superposition state; the phase oracle, where the target state is marked; and the phase estimation. The phase oracle and the phase estimation sections may be repeated as many times as desired. The last step is measuring the states of the tesseract nodes, which collapses the quantum circuit into a binary state. Figure 4 illustrates the complete quantum walk search algorithm. The entire circuit was implemented and executed using the Python programming language.

The mark section in the QuantumCircuit object, circuit, is implemented with the Python snippet:

# Mark the target pattern within the circuit

def mark(self, circuit, target):

for i in range ( len (target) - 1, - 1, - 1 ) :

if ‘0’ == target [ i ]:

circuit.x( len (target) - 1 - i)

circuit.h(3)

circuit.mct( [ 0,1,2 ] , 3)

circuit.h(3)

for i in range (0, ( len (target)):

if ‘0’ == target [ i ]:

circuit.x ( len (target) - 1 - i)

To cover all the 32768 possible input-mark, the circuit illustrated in Figure 4 was executed through a Python program. This Python program ran each execution with 1024 shots. The reader can find this Python program in the Supplementary Materials section.

### 2.5 Supplementary Materials

The data used in this study and its original program are available in GitHub at: https://github.com/dti-data/quantum-k-mer-graph.

## 3 Experiments and results

Each combination of input-mark outputs a line of data. Since the 15 bits (11 for the quantum hypercube, four for the mark) have $2^{15}$ combinations, the data collected is 32768 lines of data. Each one of those lines is a distribution of 1024 shots spread across the 16 possible states for the 2-mer qubits when the QuantumCircuit object is measured. An example of a line of output data is Table 1.

**TABLE 1:** Sample output data line with the initialization string set to “00000000000” and the mark set to “0000”.

Aux	Coin	Node	Theta	Mark	0000	0001	0010	0011	0100	0101	0110	0111	1000	1001	1010	1011	1100	1101	1110	1111
0	00	0000	0000	0000	904	5	10	10	8	3	9	13	7	8	5	14	9	5	7	7

For ease of reading, this initialization string is separated into the values used for the different quantum registers: Auxiliary (1), Coin (2), Node (4), and Theta (4). The “mark” column contains the binary values provided to the oracle as marks. The remaining 16 columns contain the frequency for each state measured at the Node register when the quantum circuit collapses.

The expected result is for the quantum walk search to hit the marked state regardless of the initialization state. Table 2 presents the number of hits for each node state when the QauntumCircuit four is initialized to the string “00000000000” and executed with 1024 shots.

**TABLE 2:** Number of hits for each vertex out of 1024 shots taken for the initialization string “00000000000”.

Aux	Coin	Node	Theta	Mark	0000	0001	0010	0011	0100	0101	0110	0111	1000	1001	1010	1011	1100	1101	1110	1111
0	00	0000	0000	0000	904	5	10	10	8	3	9	13	7	8	5	14	9	5	7	7
0	00	0000	0000	0001	3	907	7	6	9	10	5	12	7	7	8	7	7	12	12	5
0	00	0000	0000	0010	5	6	910	6	7	6	6	8	10	9	9	9	7	8	11	7
0	00	0000	0000	0011	7	5	9	911	2	9	8	9	8	5	6	10	7	8	10	10
0	00	0000	0000	0100	14	8	12	9	880	10	6	4	8	13	19	9	8	8	8	8
0	00	0000	0000	0101	7	7	4	7	7	920	8	6	8	6	11	4	3	6	12	8
0	00	0000	0000	0110	5	5	4	11	7	8	919	5	6	17	8	6	6	3	5	9
0	00	0000	0000	0111	10	3	12	13	9	13	9	898	8	7	11	7	2	5	9	8
0	00	0000	0000	1000	5	4	4	5	5	7	8	8	923	10	5	8	13	9	4	6
0	00	0000	0000	1001	5	1	11	4	7	7	13	6	8	922	9	3	8	8	8	4
0	00	0000	0000	1010	5	6	11	14	14	9	5	7	12	5	908	6	7	3	3	9
0	00	0000	0000	1011	12	9	12	7	6	10	13	11	9	8	9	893	12	3	2	8
0	00	0000	0000	1100	6	16	6	14	8	4	5	12	7	12	8	9	900	5	6	6
0	00	0000	0000	1101	4	8	11	6	7	8	10	8	6	7	13	9	11	897	11	8
0	00	0000	0000	1110	8	9	5	9	7	4	4	5	10	4	7	12	12	7	910	11
0	00	0000	0000	1111	12	5	7	10	7	8	4	4	8	6	5	6	15	2	9	916

Notably, the number of hits for each marked state is not 1024. Indeed, the quantum walk search circuit sometimes collapses to a state other than the marked state. This effect is intrinsic to quantum computing (Brassard et al., 1998). The gates applied to the qubits introduce the probability that the system will collapse into the wrong answer. Although theoretically possible, as the quantum circuits grow larger and involve more qubits, calculating the probability that a quantum circuit will collapse to a particular state becomes prohibitively complex. However, we can still shed light on the effects of an initialization state on a quantum circuit. Since the number of shots is known, Shots = 1024, and the Accuracy is directly proportional to the number of Hits for the mark when the quantum circuit is executed, the Accuracy comes to be $Accuracy=\frac{Hits}{Shots}$ . Table 3 shows the Accuracy for each marked state with the initialization string “00000000000”.

**TABLE 3:** Accuracy when executing the coined quantum walk wearch circuit with initialization string “00000000000” applied.

Mark	Accuracy
0000	0.86816
0001	0.87011
0010	0.87695
0011	0.88085
0100	0.89746
0101	0.89843
0110	0.90625
0111	0.85937
1000	0.88476
1001	0.89843
1010	0.86132
1011	0.88183
1100	0.88671
1101	0.89062
1110	0.88085
1111	0.84960

**FIGURE 5:**
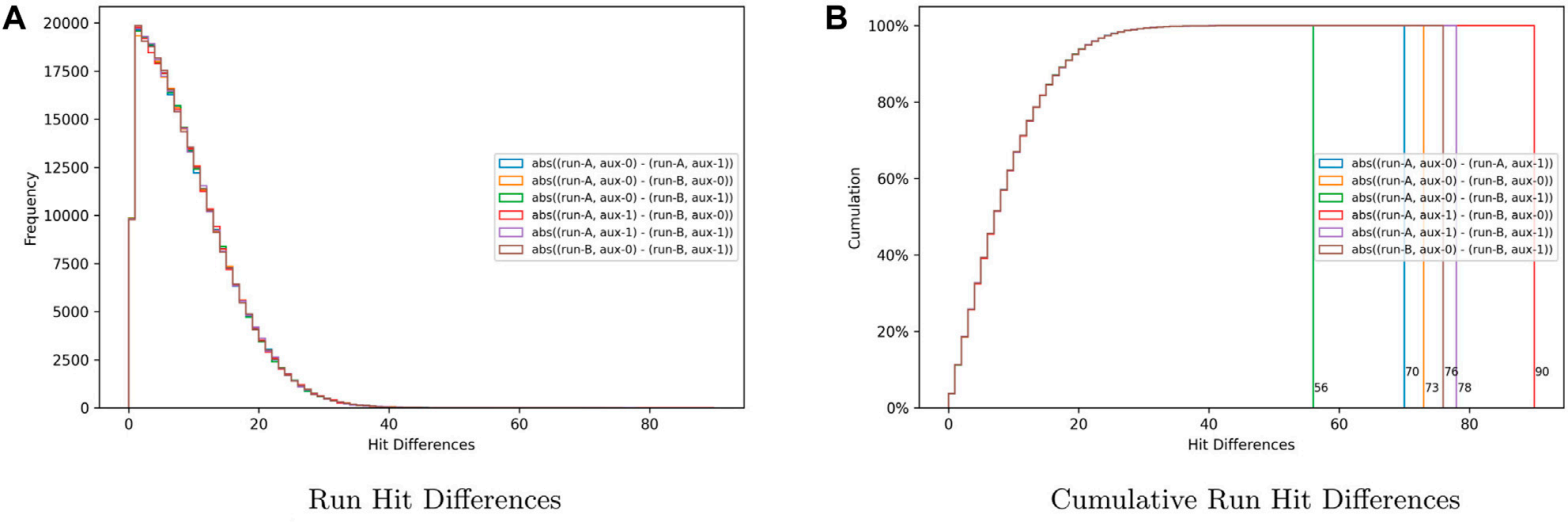
Hit differences used to determine the effect of setting the auxiliary bit to $\left|0\right\rangle$ or $\left|1\right\rangle$ . **(A)** Run hit differences. **(B)** Cumulative run hit differences.

In addition, since in quantum computing, the results are based on the probability that a circuit will collapse into a binary state for the measured qubits, the result may vary between circuit executions. One way to measure the expected variation for executions of the same circuit using a particular backend platform is to calculate the difference in hits for each state from different executions for the exact initialization string. This is the technique used in this research to determine if setting the auxiliary qubit to a $\left|0\right\rangle$ or $\left|1\right\rangle$ state using the initialization string affects the execution of quantum circuit. The results obtained are summarized in Figure 5. Figure 5A is a set of six histograms placed on the same graph to compare the differences between state hits when setting the auxiliary qubit to a $\left|0\right\rangle$ or $\left|1\right\rangle$ state in the initialization string and using that string with each of the 16 possible target states.

The differences and similarities in the hit difference distribution are readily apparent. The six distributions follow a similar skewed right distribution with slight variations, which are accounted for by the random nature of quantum computing. Figure 5B shows the cumulative distributions. This set of graphs presents the maximum difference for each distribution. Given that all the graphs display similar skewed right distributions and the mode is calculated to be 1.5 for every one of them, the conclusion is that the samples are equivalent, and, therefore, initializing the auxiliary qubit to 0 or one does not have an effect on the results when executing the quantum coined search circuit.

**FIGURE 6:**
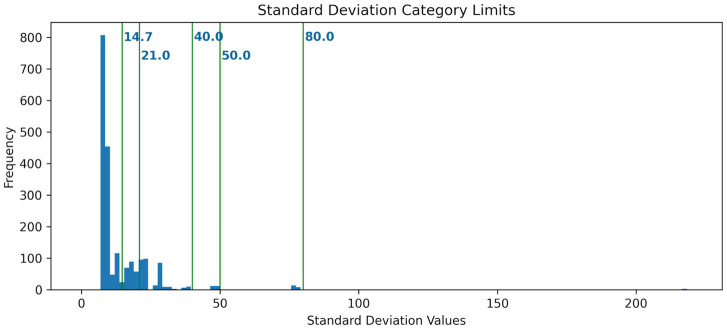
Standard deviation distributions plotted as a histogram along with arbitrary limits to organize the results for initialization string according to how randomized the results are.

Since the tesseract used for the coined quantum walk contains 16 nodes, each of which may be used as a target, each unique initialization string is used 16 times in this experiment. In addition, each execution of the quantum circuit using a particular initialization string is a 1024-size sample since the circuit execution is set to attempt 1024 shots. Also, each shot is an independent event. Therefore, calculating the standard deviation, denoted as σ , for the results obtained using an initialization string produces information that sheds light on the usability of each initialization string. Figure 6 presents the distribution of standard deviation values for the distribution of hits for the collapsed states.

The standard deviation measures how close the number of hits for a state is to the expected value of 64 hits (1024 shots/16 possible states). The smaller the value of σ for the hit distribution, the closer each state gets to getting 64 hits. Therefore, it also measures how random the circuit produces the hits for a given initialization string. As σ increases, the hit distribution is skewed into a few states. This means that the greater the standard deviation for the results for a given initialization string is, the better defined a pattern within the results is.

Classifying the initialization strings based on the standard deviation values aids in visualizing the patterns for the hit distributions. To leverage this analysis technique, let us define six arbitrary categories such that σ takes the values as shown in Table 4.

**Table 4:** Hit distribution categories based on the standard deviation calculated for the hit distribution for each initialization string.

Random: σ < 14.7 Normal distribution, mode near the expected value, 64.
Emerging: 14.7 ≤ σ < 21.0 Single modal distribution, mode diverts from 64.
Weak: 21.0 ≤ σ < 40.0 Multimodal distribution, patterns are discernible.
Complex: 40.0 ≤ σ < 50.0 Multimodal Patterns are readily distinguished.
Clear: 50.0 ≤ σ < 80.0 Multimodal with nearly disconnected modes.
Strong: 80.0 ≤ σ The distribution is bimodal with disconnected modes.

**FIGURE 7:**
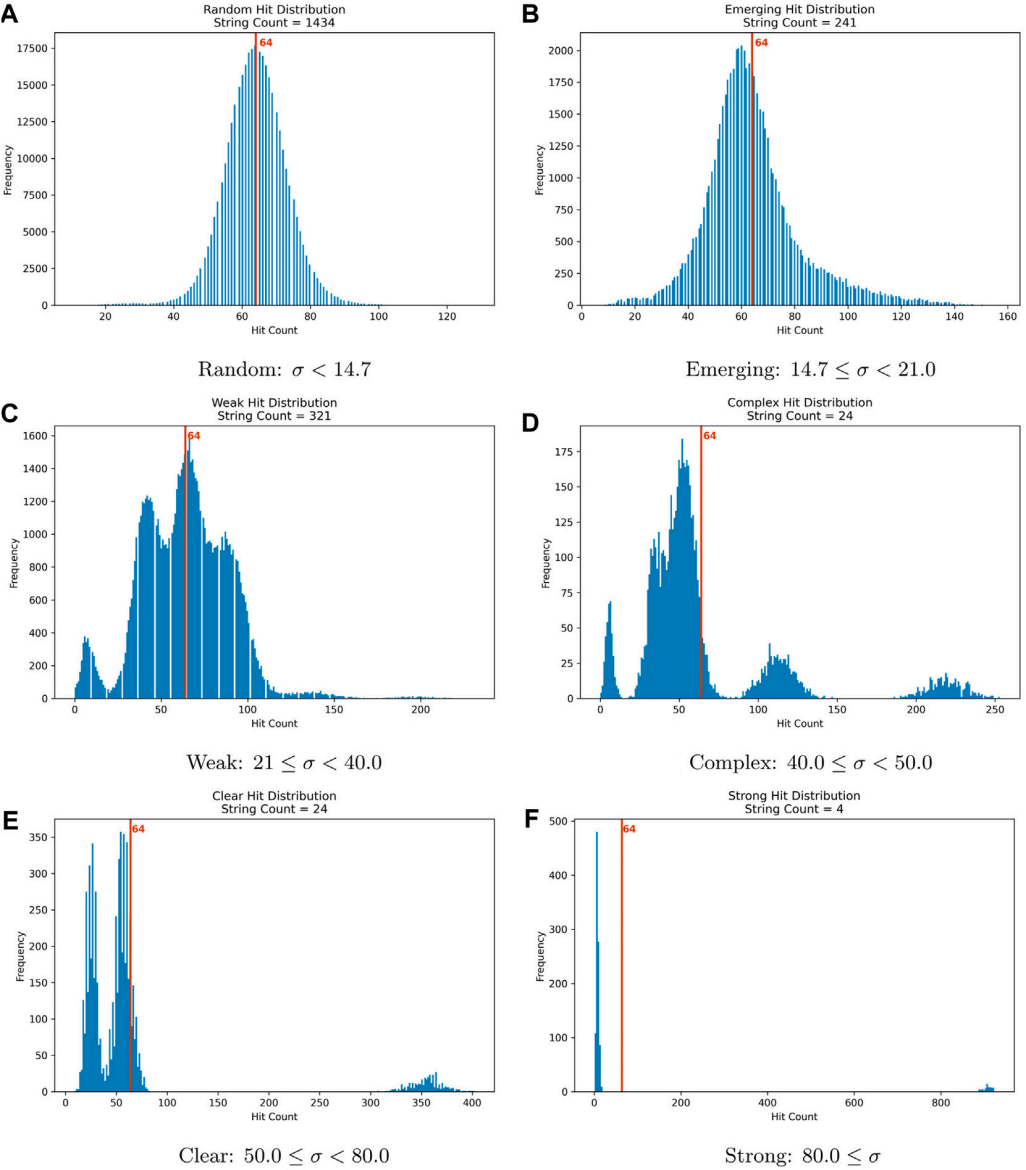
Hit distributions for the categories in Table 4 based on the standard deviation of each execution with 1024 shots. **(A)** Random: σ < 14.7; **(B)** Emerging 14.7 ≤ σ < 21.0; **(C)** Weak: 21 ≤ σ < 40.0; **(D)** Complex: 40.0 ≤ σ < 50.0; **(E)** Clear: 50.0 ≤ σ < 80.0; **(F)** Strong: 80.0 ≤ σ.

The set of Figure 7 displays the six resulting hit distributions with the limits for σ defined in Table 4.

The standard deviation, σ , for the string “00000000001” results is 28.53, which places it into the Weak category. However, finding a marked state is an interpretation exercise. An interpreter may use the fact that the marked state is being avoided since it gets fewer hits than the rest to find the marked item. In addition, although the standard deviation for the hit distribution for this string is low, the accuracy for finding the marked state trough avoidance would be much higher.

The results for “00000110010” have a standard deviation of 49.26 which belongs to the Complex category.

## 4 Discussion

Quantum computing is a relatively new but rapidly evolving field. Currently, the manipulation of quantum circuits is done at the gate level. This activity requires detailed knowledge of quantum computing. While efforts are underway to ease the expertise requirements through software stacks, executing quantum circuits may not produce the expected results. Take, for instance, the experiments performed in this research. Although the inputs and marks were applied to the circuit using the same techniques, the results are inconsistent. This finding prompts us to dissect the quantum circuit and analyze what happens deeper into the different execution levels to leverage those phenomena and the information density from the quantum hypercube to implement faster k-mer searching techniques. The effort to organize and summarize the data in the categories presented in Table 4, is to reference the outcomes and focus further research, beyond the scope of the current work, on the different behaviors prompted by the inputs.

One of the surprising outcomes of the experiments was that only four initialization strings produced a “Strong” output pattern using the coined quantum walk as is. This outcome is the expected behavior. The marked k-mer in the hypercube is hit the most times. Those four initialization strings are: “00000000000”, “00011110001”, “10000000000”, “10011110001”. The bit in position 10 is loaded into the auxiliary qubit which has no effect. Therefore, the set is reduced to “0000000000”, and “0011110001”.

Another finding is that While the quantum circuits, when implemented with superposition, may leverage the parallel processing of a quantum device, changing even the initial state of a qubit may change the quantum circuit behavior so dramatically that when measured, it collapses to a random state. This is the output of 1434 initialization strings with a hit distribution with a standard deviation less than 14.2; therefore, the results are “Random”, Figure 7A. This count is already more than half of the possible initialization strings.

The “Emerging” category is close to having a normal distribution but with some distortions. Some states get hits that diverge significantly from the expected value but more is needed to establish a pattern.

The “Weak” patterns already show an accumulation of hits around values other than 64. One feature in this category is that the quantum search establishes a pattern on the marked state by hitting it with the least frequency, as is the case with the initialization string “00000000001” as shown in Table 5. This effect may be useful in finding the marked state through avoidance since “finding” is an interpretation exercise.

**TABLE 5:** Results for executing the coined quantum walk search on a 2-mer hypercube with initialization string “00000000001”.

aux	Coin	Node	Theta	Mark	0000	0001	0010	0011	0100	0101	0110	0111	1000	1001	1010	1011	1100	1101	1110	1111
0	00	0000	0001	0000	6	97	85	38	91	40	42	75	92	40	42	89	57	93	94	43
0	00	0000	0001	0001	86	11	42	81	41	95	89	52	50	89	91	42	74	54	43	84
0	00	0000	0001	0010	97	28	6	99	30	80	86	33	39	89	104	55	79	50	51	98
0	00	0000	0001	0011	48	80	68	6	64	50	42	98	96	49	59	100	41	82	98	43
0	00	0000	0001	0100	82	29	43	93	7	91	89	42	45	99	89	42	83	40	50	100
0	00	0000	0001	0101	45	82	96	50	95	6	32	87	91	40	45	87	53	84	99	32
0	00	0000	0001	0110	43	118	90	42	93	35	7	100	74	49	46	88	33	83	77	46
0	00	0000	0001	0111	86	35	40	83	46	86	92	6	41	88	91	54	86	48	48	94
0	00	0000	0001	1000	97	50	42	83	44	83	81	48	6	78	100	38	87	40	50	97
0	00	0000	0001	1001	49	85	83	44	89	39	35	86	87	9	42	90	41	95	95	55
0	00	0000	0001	1010	43	81	89	36	103	54	48	78	81	38	6	97	42	93	91	44
0	00	0000	0001	1011	89	50	51	86	42	104	92	49	32	91	80	3	89	35	31	100
0	00	0000	0001	1100	49	77	87	50	106	29	54	74	107	40	44	90	2	83	93	39
0	00	0000	0001	1101	94	50	45	81	40	87	79	47	31	95	83	35	99	5	50	103
0	00	0000	0001	1110	82	51	44	85	39	88	80	36	46	91	92	42	79	54	8	107
0	00	0000	0001	1111	55	80	97	36	83	46	51	96	78	44	36	91	34	92	98	7

The “Clear” category displays hits consolidating on the marked state, just as in the Strong category, but the hit count is far from being 100%.

The category “Complex” is named as such based on the patterns displayed on the hit distribution. The hits are accumulated around the marked state, but the node with the binary inverse of the marked state is also avoided. Even more, the circuit hits other states, forming a complex pattern. As shown in Table 6.‘’ The input-mark combinations in this category output are intriguing and may be the subject of deeper studies.

**TABLE 6:** Results for executing the coined quantum walk search on a 2-mer hypercube with initialization string “00000110010”.

aux	Coin	Node	Theta	Mark	0000	0001	0010	0011	0100	0101	0110	0111	1000	1001	1010	1011	1100	1101	1110	1111
0	00	0011	0010	0000	232	55	43	105	60	33	38	66	53	29	37	42	108	54	64	5
0	00	0011	0010	0001	42	226	123	49	31	50	52	37	42	50	59	37	57	107	4	58
0	00	0011	0010	0010	50	106	240	51	38	57	56	22	30	57	43	37	52	7	118	60
0	00	0011	0010	0011	115	56	57	233	65	30	31	39	57	44	28	49	7	48	56	109
0	00	0011	0010	0100	51	31	33	44	216	51	71	98	118	59	48	7	67	34	42	54
0	00	0011	0010	0101	28	55	62	33	55	234	110	53	63	103	3	54	30	43	54	44
0	00	0011	0010	0110	38	52	46	37	58	100	220	55	37	6	121	55	45	58	57	39
0	00	0011	0010	0111	48	41	30	56	97	64	52	246	1	72	44	102	52	38	33	48
0	00	0011	0010	1000	62	33	35	48	110	57	61	3	233	56	53	110	51	22	25	65
0	00	0011	0010	1001	37	47	63	41	73	107	4	56	50	214	102	53	45	59	43	30
0	00	0011	0010	1010	30	59	41	36	56	7	128	67	67	96	214	54	34	53	47	35
0	00	0011	0010	1011	46	32	31	51	5	64	35	125	122	53	52	218	52	40	42	56
0	00	0011	0010	1100	126	51	56	7	52	30	26	55	55	28	42	64	214	65	42	10011
0	00	0011	0010	1101	46	119	6	60	45	52	51	38	48	65	46	30	49	221	104	44
0	00	0011	0010	1110	45	6	116	47	29	71	45	37	36	58	58	28	62	109	213	64
0	00	0011	0010	1111	5	43	50	117	49	46	32	51	48	45	30	55	119	58	53	223

The presented categories show that there is much to be researched and developed for the coined quantum walk search on a 2-mer quantum hypercube to be practical. In theory, the quantum hypercube has an exponential information density. The fact that a quantum N-dimensional hypercube can represent $2^{N}$ vertices with N qubits is awe-inspiring. This information density is even more impressive when compared to the classical bits necessary to define the vertices for an N-dimensional hypercube, which are $2^{N}*N$ . As technology allows for larger quantum computers and quantum algorithm design becomes reusable, developing techniques to exploit the features of a quantum hypercube as a k-mer graph will become essential in our quest to make sense of the vast information nature has in store for us. While the outcomes produced in the experiments have little use in practical applications, the discoveries made bring us closer to applying quantum computing to bioinformatics.

## 5 Conclusion

Encoding binary data into a quantum computer is possible through the initialization string and marking the desired quantum states. Thus, it is possible to encode DNA sequences into such a device. Once the hypercube is built with marked DNA k-mer fragments, the coined quantum walk search is able to return useful results on some instances. However, only some initialization strings output useful repeatable patterns from which information may be extracted.

One limitation of the coined quantum walk search on a 2-mer hypercube is that it is not a universal search technique. The search design has to be adapted to the specific input string. The wide difference in results supports this assertion. Therefore, while a quantum computer can represent an N-dimensional hypercube with N qubits and exploit parallelism in searching, a substantial limitation is that the circuit does not behave consistently for all input-mark combinations.

Another limitation is that the k-mers in the hypercube are of fixed length, in this research, 2-mer, as the hypercube was created. If a different size of k-mer is required, a new hypercube needs to be constructed.

Since quantum computing is still a young field, much research is being done to explore and demonstrate its usefulness. One possible improvement beneficial for adopting this powerful paradigm is developing high-level methods or functions that behave consistently in the face of different inputs.

## Data Availability

The raw data supporting the conclusions of this article will be made available by the authors, without undue reservation.
